# The Effect of an Aqueous Extract of *Teucrium polium* on Glutathione Homeostasis In Vitro: A Possible Mechanism of Its Hepatoprotectant Action

**DOI:** 10.1155/2010/938324

**Published:** 2010-03-23

**Authors:** Stella Shtukmaster, Predrag Ljubuncic, Arieh Bomzon

**Affiliations:** Department of Pharmacology, Bruce and Ruth Rappaport Faculty of Medicine, Technion-Israel Institute of Technology, P.O. Box 9649, Haifa 31096, Israel

## Abstract

*Background*. *Teucrium polium* is used in Arab traditional medicine to treat liver diseases. Glutathione is an important intracellular antioxidant, and intrahepatic glutathione levels are depleted in liver diseases. *Hypothesis and Aim*. This investigation tested the hypothesis that aqueous extracts of *T. polium* maintains intracellular glutathione levels by augmenting glutathione peroxidase and glutathione reductase activity in cultured hepatocytes. *Methods*. The effects of increasing concentrations (0.01–1 mg/mL) of aqueous extract of *T. polium* were assessed in cultured HepG2 cells following 24 hours incubation on (1) cellular integrity using (a) the Trypan blue exclusion assay, (b) the [di-methylthiazol-2yl]-2,5-diphenyl-tetrazoliumbromide (MTT) assay, and (c) the lactate dehydrogenase (LDH) assay; (2) glutathione redox state; and (3) glutathione peroxidase and glutathione reductase activities using a repeated measures experimental design. *Results*. At concentrations of 0.375 mg/mL and 0.5 mg/mL, the extract increased the intracellular levels of total and reduced glutathione and had no effect on the intracellular amounts of oxidized glutathione. The extract had no effect on glutathione peroxidase and glutathione reductase activities. *Conclusion*. These data indicate that the mechanism of the hepatoprotective action of aqueous extracts of *T. polium* may be, in part, due to augmenting intracellular glutathione levels.

## 1. Introduction

In traditional Arab medicine, an extract of *Teucrium polium* (Polygermander), a member of Lamiaceae family and widely distributed in the hills and deserts of Mediterranean countries, is used to treat liver disease, hypertension, and diabetes [1]. The extract is also used as an antiemetic, an antispasmodic, an antiinflammatory, an antipyretic, an analgesic, and an anti-carminative, and in the treatment of hyperlipidemia and peptic ulcers [2]. 

The therapeutic benefits of *T. polium* extracts are usually attributed to their ability to suppress oxidative processes. For example, Suboh and colleagues [3] showed that an alcoholic extract of *T. polium* could suppress hydrogen peroxide-induced lipid peroxidation in red blood cells in a concentration-dependent manner. Previously, we reported that an aqueous extract of *T. polium* suppressed iron (Fe²^+^)—induced lipid peroxidation in rat liver homogenates at concentrations that were not toxic to cultured hepatocytes [4]. We also reported that an aqueous extract of the plant could scavenge the superoxide anion and hydroxyl radical in a concentration-dependent manner and could inhibit other oxidative processes in vitro, such as the oxidation of *β*-carotene and plasma, as well as chelating iron [5].

There is evidence that the liver is oxidatively stressed in hepatic disease due to ongoing oxidative processes and depletion of intrahepatic glutathione levels [6, 7]. Based on the fact that extracts of *T. polium* are still widely used to treat liver disease in traditional medicine, it is reasonable to assume that the possible mechanisms of the extract's therapeutic actions are due to the extract's ability to suppress oxidative processes and maintain endogenous antioxidant levels. However, there are no laboratory data on the extract's effects on endogenous antioxidants, such as glutathione. Therefore, we designed a study to assess the effects of an aqueous extract of *T. polium* on intracellular glutathione homeostasis. In addition, we also assessed the effects of increasing concentrations of an aqueous extract on cellular viability and integrity of cultured hepatocytes, and the results are presented in the current communication.

## 2. Materials and Methods

### 2.1. Plant Material and Extract Preparation

Leaves and the stems of *T. polium* were collected during spring (May-June) from the hills of the Galilee region of Israel. After collection, the plant parts were dried for 7–10 days in the shade at room temperature. They were then ground and the powder was stored in cloth bags at 5°C until the aqueous extract was prepared. For this purpose, the dried plant material (25 g) was stirred in 250 mL of distilled water for 15 min at 95°C, followed by rapid filtration first by a crude cellulose filter and then using Whatman #1 filter paper. The average w/w yield was 11.5%. The resulting solution was freeze-dried and the powder was stored at −18°C in a desiccant until required.

### 2.2. Chemical and Reagents

All culture media and reagents for the assays that involved cultured hepatocytes were purchased from Biological Industries Ltd., Beit Haemek, Israel. All other chemicals and reagents were of the highest purity grade and purchased from the Sigma Chemical Co., St. Louis, MO, USA.

### 2.3. Cell Culture

The effects of an aqueous extract of *T. polium *on cellular integrity, glutathione redox state, and the activities of GPx and GR were assessed in cultured HepG2 cells. HepG2 cells are derived from human hepatoblastoma and retain many of the differentiated features of mature hepatocytes [8]. The cells were grown in Roswell Park Memorial Institute medium (RPMI 1640) that contained glucose (2 g/L) and supplemented with 10% fetal calf serum, 10000 U/mL penicillin, 10 mg/mL streptomycin, 1% glutamine, 1% Hepes buffer solution, pH 7.4, and maintained in a humidified atmosphere of 95% O_2_ : 5% CO_2_ at 37°C. At 80% confluence, cells were trypsinized, centrifuged (1700 rpm for 5 minutes at room temperature), resuspended in fresh medium, and plated in microtiter wells (2 × 10^4^ cells/well) or in six-well dishes (10^6^ cells/well). After attachment, they were incubated in serum-free medium to which 0.01–1 mg/mL extract was added and kept for 24 hours at 37°C.

### 2.4. Assays of Cellular Integrity

Three different assays were used to assess the effect of the extract on cellular integrity: (a) the Trypan blue exclusion test to determine cell viability, (b) the [di-methylthiazol-2yl]-2,5-diphenyl-tetrazoliumbromide (MTT) assay to monitor mitochondrial respiration; (c) the lactate dehydrogenase (LDH) assay to assess plasmalemma integrity.

#### 2.4.1. Trypan Blue Exclusion Assay

The Trypan blue exclusion assay is widely used to determine cellular integrity [9, 10]. HepG2 cells (2 × 10^6^ cells/well) were incubated with 0.01 – 1 mg/mL plant extract for 24 hours at 37°C. The cells were then exposed to the dye and the number of cells that took up the dye was counted in a hemocytometer. The proportion of dye-containing cells to cells that did not take up the dye was then calculated. The experiment was repeated between 7–10 times.

#### 2.4.2. MTT Assay

The MTT assay is a test of metabolic competence and assesses mitochondrial performance [9, 10]. It is a colometric assay that relies on the conversion of yellow tetrazolium bromide (MTT) to the purple formazan derivative by mitochondrial succinate dehydrogenase in viable cells [11]. Briefly, HepG2 cells (2 × 10^4^ cells/well) were first incubated with 0.01–1 mg/mL plant extract for 24 hours at 37°C. The cells were then incubated in serum-free medium to which MTT (0.5 mg/mL, 10 *μ*L) was added. Following 3.5 hours incubation, 100 *μ*L acidic isopropanol (0.04–0.1 N HCl in absolute isopropanol) were added to dissolve the formazan crystals and the absorbance was determined in an ELISA reader at 570/650 nm. The number of metabolically competent cells was determined as the ratio (expressed as a percentage) of absorbance of treated cells to untreated cells that served as control. The experiment was repeated nine times.

#### 2.4.3. LDH Assay

The presence of the cytosolic enzyme, LDH, in the cell culture medium is indicative of cell membrane damage [9, 10]. Briefly, HepG2 cells (2 × 10^4^ cells/well) were first incubated with 0.1–1 mg/mL plant extracts for 24 hours at 37°C. Upon completion of the incubation, 50 *μ*L of the upper medium were collected from each well. The untreated cells were then lysed with a cell lysis solution for 40 minutes at room temperature and the lysate collected. LDH activity was measured using CytoTox 96 Nonradioactive Cytotoxicity Assay Kit (Promega, WI, USA), in accordance with manufacturer's instructions. The percent of LDH released from the cells was determined using the formula: (absorbance of supernatant)/(absorbance of supernatant + absorbance of lysate) × 100. The experiment was repeated seven times.

### 2.5. Determination of Total Intracellular Amount of Glutathione

HepG2 cells were seeded and grown in cell flasks under the identical conditions which have already been described. At 80% confluence, cells were trypsinized, centrifuged (1700 rpm for 5 minutes at room temperature), resuspended in fresh medium, and plated in six-well dishes (10^6^ cells/well). After attachment, the medium was replaced and cells were incubated in fresh serum-free medium containing the extract for 24 hours at 37°C. The nontreated cells served as the control. At the end of incubation period, the cells were washed three times with Dulbecco's phosphate buffered saline (PBS). After washing, the cells were scraped into 1 mL PBS. To extract cellular GSH, the cells were then dispersed using a sonicator by two 20 sec bursts. An aliquot of sonicate was taken for protein determination [12]. The remainder of sonicate was immediately acidified with 5% sulfosalicylic acid (2 : 1 v/v) to prevent spontaneous oxidation of GSH. After standing for 10 m on ice, the sonicate was centrifuged at 10000 rpm for 10 min at 4°C to remove the denaturated proteins. The resultant supernatants were transferred into 1.7 mL plastic tubes and the acidified samples were frozen at −70°C until use that was always less than one week. 

GSH levels were determined using the DTNB-GSSG reductase recycling assay [13] with minor modifications. Before measurement of GSH, the sample was thawed and back titrated to pH 7.0 with 0.2 N NaOH. Total GSH content (the sum of the reduced and oxidized forms of glutathione) and the oxidized form (GSSG) were measured separately. The GSH assay was performed in cuvette that contained 0.01 M sodium phosphate buffer (pH 7.5), 5 mM EDTA, 10 mM 5,5′-dithio-bio-2-nitrobenzoic acid (DTNB), 2 mM nicotinamide adenine dinucleotide phosphate (NADPH), 10 U/mL glutathione reductase, and approximately 100 *μ*g cell protein in a final volume of 1 mL. The reaction kinetics were followed spectrophometrically at 412 nm for 5 minutes by monitoring the increase in absorbance. 

For determination of GSSG content, the identical DTNB recycling assay was performed after alkylation of the SH groups of reduced glutathione by 10 mM N-ethylmaleimide (NEM) in order to remove reduced glutathione from the reaction. To avoid the adverse effects of excess N-ethylmaleimide in the assay used to determine GSSG levels, excess NEM was removed by separation on a Sep-Pak (C18) column (Sigma Chemical Corp., MI, USA). The increments in absorbance at 412 nm were converted to GSH and GSSG concentrations using a standard curve (0–2.5 nmol GSSG). The results were expressed in nmol/mg protein. The experiment was repeated eight times in which duplicate GSH and GSSG determinations were done.

### 2.6. Determination of Glutathione Peroxidase (GPx) Activity

HepG2 cells were seeded and grown in cell flasks and then in six-well dishes (10^6^ cells/well) under the identical conditions that have already been described. At the end of incubation period HepG2 cells were washed and scraped into 1 mL PBS and then were sonicated in the same manner as were prepared for glutathione determination. An aliquot (100 *μ*L) of the sonicate was taken for protein determination [12]. The remainder of sonicate was then used to determine the activities of the enzymes.

The enzyme, GPx, detoxifies peroxides in the cell. Due to the fact that peroxides can decompose to form highly reactive radicals, GPx plays a vital role in protecting the cell from free radical-induced damage, particularly lipid peroxidation. The enzyme also catalyzes the reduction of hydrogen peroxide and organic peroxides (R–O–O–H) to water and corresponding stable alcohols (R–O–H), respectively, using reduced glutathione as a source of reducing equivalents. When oxidized glutathione is produced upon reduction of organic peroxide by GPx, it is recycled to its reduced state by GR with oxidation of NADPH to NADP^+^. The process of NADPH oxidation is accompanied by a decrease in absorbance at 340 nm thereby providing a spectrophotometric means for monitoring the activity of GPx. GPx was assayed using t-butylhydroperoxide (t-BOOH) as a substrate [14]. The assay was performed in cuvette that contained 1 M Buffer Tris-HCl + 5 mM EDTA (pH 8.0), 0.1 M GSH, 2 mM NADPH, 10 U/mL glutathione reductase, sample, and t-butylhydroperoxide diluted 1 : 1000 with distilled water in a final volume of 1.0 mL. The decrease in absorbance, reflecting the oxidation of NADPH which directly proportional to the GPx activity in the sample, was followed at 340 nm. Results were expressed as units of GPx activity/mg cell protein. The experiment was repeated ten times.

### 2.7. Determination of Glutathione Reductase (GR) Activity

The enzyme, GR, catalyzes the reduction of GSSG to GSH and is essential for the glutathione redox cycle in order to maintain adequate levels of reduced cellular GSH. During the reduction of GSSG by glutathione reductase, one molecule of NADPH is consumed for each molecule of GSSG reduced. Therefore, the reduction of GSSG by GR can be determined by the measurement of the consumption of NADPH. The activity of GR was assayed using the method that was described by Carlberg and Mannervik [15] with minor modifications. The GR assay was performed in a cuvette that contained 1 M Tris-HCl buffer + 5 mM EDTA (pH 8.0), 0.033 M GSSG, 2 mM NADPH, and a sample in a final volume of 1.0 mL. The decrease in absorbance, which reflects the oxidation of NADPH during reduction of GSSG by GR present in the sample, was monitored spectrophotometrically at 340 nm. Results were expressed as units of GR activity/mg cell protein. The experiment was repeated ten times.

### 2.8. Statistical Analysis of the Data

The sample size for each experiment was determined by power analysis arbitrarily set at 80% in order to detect an effect at 5% probability using Statemate version 1 (GraphPad Software, Inc., San Diego, CA, USA). The effect of the varying concentrations of the extract was assessed on the various study parameters using a repeated measures experimental design. The data of the effects of an aqueous extracts of *T. polium* on cell viability, the glutathione redox state and, and the activities of GPx and GR were analyzed by one-way analysis of variance (ANOVA) with a Dunnett's posttest. All data are expressed as mean ± standard deviation.

## 3. Results

### 3.1. The Effect of an Aqueous Extract of *T. polium* on the Integrity of HepG2 Cells

First, we examined the effect of the increasing concentrations of extract of *T. polium* on the viability of cultured HepG2 cells using the Trypan blue exclusion test and found that the extract had no effect on cellular viability following 24 hours incubation with the extract (data not shown).

**Figure 1:**
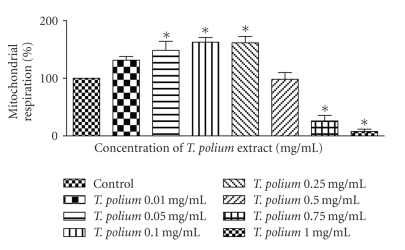
Effect of increasing concentrations of an aqueous extract of *T. polium* on mitochondrial respiration in HepG2 cultured cells using the MTT assay. Data are presented as mean ± standard deviation. Sample size (*n*) = 9. *p < 0.01 represents the significance of the difference from the control. All data are depicted as a percentage with respect to control (100% mitochondrial respiration).

We then used the MTT assay to assess the effect of the identical concentrations of the aqueous extract on the mitochondrial respiration of cultured HepG2 cells. At the lower concentrations, 0.05–0.25 mg/mL, the aqueous extract boosted mitochondrial respiration (*p* < 0.01). At the concentrations of 0.01 mg/mL and 0.5 mg/mL, the extract had no effect on mitochondrial respiration. At higher concentrations 0.75–1 mg/mL, the extract inhibited mitochondrial respiration (*p* < 0.01) (Figure 1). 

**Figure 2:**
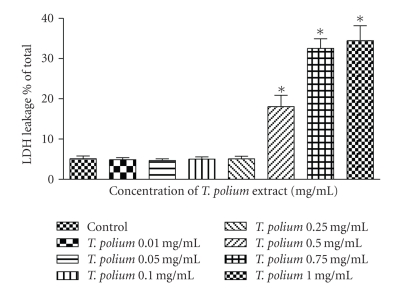
Effect of increasing concentrations of an aqueous extract of *T. polium* on membrane integrity in HepG2 cultured cells using the LDH assay. Data are presented as mean ± standard deviation. Sample size (*n*) = 7. *p < 0.01 represents the significance of the difference from the control. All data are presented as a percentage of the total (100%).

The LDH assay was then used to determine the effect of the extract on the integrity of the plasmalemma of cultured HepG2 cells. At the low concentrations, 0.01–0.25 mg/mL, the extract had no effect on the LDH leakage following 24 hours incubation. At higher concentrations 0.5–1 mg/mL, the extract increased cellular LDH efflux (*p* < 0.01) (Figure 2). 

Based on these findings, three concentrations of the extract, namely, 0.25 mg/mL, 0.375 mg/mL, and 0.5 mg/mL of the extract, were then used to evaluate the effect of the extract on intracellular glutathione homeostasis.

### 3.2. The Effect of an Aqueous Extract of *T. polium* on Total Glutathione Levels in Cultured HepG2 Cells

**Figure 3:**
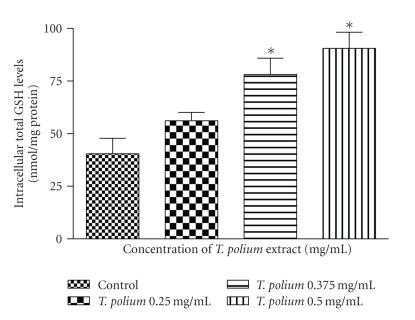
Effect of a 24-hour exposure of an aqueous extract of *T. polium* on total glutathione levels in HepG2 cultured cells. Data are presented as mean ± standard deviation. Sample size (*n*) = 8. *p < 0.01 represents the significance of the difference from the control.

**Figure 4:**
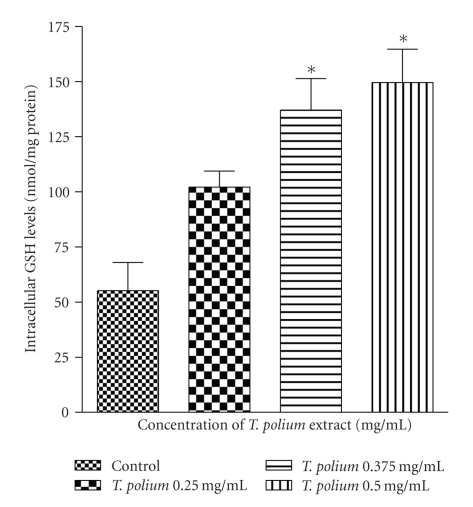
Effect of a 24-hour exposure of an aqueous extract of *T. polium* on reduced glutathione (GSH) levels in HepG2 cultured cells. Data are presented as mean ± standard deviation. Sample size (*n*) = 8. *p < 0.01 represents the significance of the difference from the control.

Over the 0.375–0.5 mg/mL concentration range, the extract significantly increased (*p* < 0.01) the intracellular levels of total (Figure 3), reduced glutathione (Figure 4), and had no effect on the amount of GSSG (data not shown) in HepG2 cells. The results of total and reduced glutathione are similar because intracellular glutathione is found almost exclusively in its reduced form.

Due to the fact that the aqueous extract increased the reduced glutathione levels without affecting oxidized glutathione levels over the 0.375–0.5 mg/mL concentration range, the GSH/GSSG ratio increased.

### 3.3. The Effect of an Aqueous Extract of *T. polium* on the Activities of Glutathione Peroxidase and Glutathione Reductase

**Figure 5:**
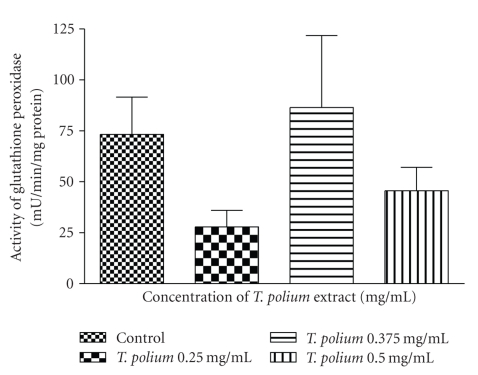
Effect of a 24-hour exposure of an aqueous extract of *T. polium* on glutathione peroxidase activity in HepG2 cultured cells. Data are presented as mean ± standard deviation. Sample size (*n*) = 10.

**Figure 6:**
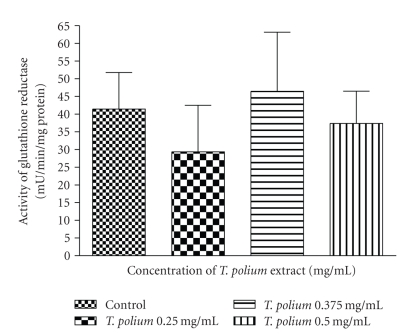
Effect of a 24-hour exposure of an aqueous extract of *T. polium* on glutathione reductase activity in HepG2 cultured cells. Data are presented as mean ± standard deviation. Sample size (*n*) = 10.

Over the 0.25–0.5 mg/mL concentration range, we found that the extract had no effect on the activities of GPx (Figure 5) and GR (Figure 6) after a 24-hour exposure.

## 4. Discussion

The aim of this work was to assess the effects of an aqueous extract of *T. polium* on the homeostasis of intracellular glutathione. In this study, we found that an aqueous extract of* T. polium* had no effect on cellular integrity at low (0.01–0.25 mg/mL) concentrations. At higher concentrations (0.75–1 mg/mL), the extract was toxic to cells because it inhibited mitochondrial respiration and increased cellular LDH efflux. At concentrations of 0.375 mg/mL and 0.5 mg/mL, the extract significantly increased the intracellular levels of total and reduced glutathione and had no effect on intracellular amounts of oxidized glutathione. Since the extract increased the intracellular levels of reduced glutathione without affecting oxidized glutathione levels, the GSH/GSSG ratio increased. At the same time, we found that the extract had no effect on the activities of GPx and GR. 

We found that the maximal nontoxic concentration of the extract of *T. polium* was between 0.25 mg/mL and 0.5 mg/mL. Therefore, we then examined the effect of the extract over this concentration range on intracellular glutathione homeostasis. We observed that an aqueous extracts of *T. polium* at the concentrations of 0.375 mg/mL and 0.5 mg/mL increased the intracellular total and reduced glutathione levels without affecting the intracellular levels of oxidized glutathione. Accordingly, we presumed that the extract was capable of increasing the activities of GPx and GR, which resulted in enhanced intracellular glutathione cycling. However, we did not see such an effect on the activities of the two enzymes. It is well documented that the end product of glutathione biosynthesis is reduced glutathione [16, 17]. In view of our findings that the extract had no effect on the activities of GPx and GR reductase, we suggest that *T. polium* enhances intracellular glutathione levels by promoting the glutathione biosynthetic pathway. Further experiments on the effect of the extract on the activity of glutathione synthetase are needed to verify this suggestion.

The possible antioxidant mechanism of action of the extract includes its ability to scavenge reactive oxygen species or/and enhance endogenous antioxidants levels. In present study we found that *T. polium* has an ability to augment the levels of an important intracellular antioxidant. Kadifkova-Panovka and colleagues [18] reported that rats that the plasma GSH levels of rats that were pretreated with a *T. polium* extract before inducing cirrhosis with carbon tetrachloride were partially depleted when compared to the plasma levels in untreated CCl_4_-induced cirrhotic rats. Their finding suggests that *T. polium* extract has a possible hepatoprotective effect. Accordingly, this might be the underlying reason why this plant is used to treat liver disease in traditional Arab medicine. 

Although we did not try to identify the antioxidant bioconstituents of *T. polium* in this study, Rizk et al. reported that the aerial parts of the plant are rich with flavonoids [19]. Flavonoids exhibit many biological activities, such as antitumoral, antiischemic, antiallergic, anti-hepatotoxic, and antiinflammatory activities, and many of these activities are attributed to their antioxidant potential [20–22]. Flavonoids and other phenolic compounds have been reported as scavengers of reactive oxygen species and inhibitors of lipid peroxidation [23]. There are some reports of the effects of some flavonoids on glutathione. Zhang demonstrated that simultaneous supplementation with quercetin restored GSH content after oxidative damage in cultured spermatogonial cells [24]. Other researchers demonstrated that flavonoids could stimulate the transcription of the gene(s) that are responsible for intracellular GSH synthesis [25, 26]. Flavonoids are the not only constitutes of *T. polium*. *T. polium* also contains various diterpenoids [27] and furanoneoclerodane diterpenoids which are known hepatotoxins [28–30]. Although diterpenoids can be toxic to cells, we could not found any evidence of such an activity at low concentrations of the *T. polium* extract that was used in this study. Nevertheless, we found that *T. polium* is toxic to cells at higher concentrations, and such compounds could be the bioactive constituents that are responsible for the observed cytotoxicity at the higher concentrations. Furthermore, it should not be overlooked that we investigated the effects of the extract in a cell culture system in which the toxic diterpenoid concentration would increase as the extract concentration increases. We propose this as the reason for the inhibition of mitochondrial respiration and increase of cellular LDH efflux at the higher concentrations of aqueous extract of *T. polium*. Therefore, further investigations are needed in order to clarify the mechanism of the hepatoprotectant action of flavonoids and hepatotoxic action of dipertenoids in the various types of *T. polium* extracts whose flavonoid and dipertenoid levels may vary according to the location and season of plant collection, and the preparation and type of extract. 

Our results also provide some insight into the controversy on the safety and hepatoprotective and hepatotoxic potential of *T. polium* extracts because they highlight the delicate balance between the beneficial and toxic effects of a *T. polium* extract on glutathione homeostasis and cellular integrity. There are now many reports on *T. polium*-induced hepatotoxicity with sometimes severe and fatal complications in humans [30–37]. This hepatotoxicity has been confirmed in laboratory animals [27, 28, 38, 39] and cultured hepatocytes [4, 5]. In this study, we demonstrated that cellular integrity is unaffected at low extract concentrations, and continues to be unaffected despite nonsignificant increases in intracellular glutathione levels as extract concentration increases. Further modest increases in extract concentration cause an additional increase in intracellular glutathione levels and appearance of impaired cellular integrity (reduced mitochondrial respiration and increased LDH leakage). In other words, the concentrations at which the extract increases intracellular glutathione levels coincide with those that can impair cellular integrity. 

In conclusion, our data indicates that, at low concentrations, an aqueous extract of *T. polium* is not toxic and the mechanism of the hepatoprotective action of aqueous extract may be, in part, due to enhancement of intracellular glutathione levels. The mechanism of this increase is not due to the extract accelerating intracellular glutathione recycling, but could be due to an effect on glutathione biosynthesis.
